# Behavioral dissociation between emotional and non-emotional facial expressions in congenital prosopagnosia

**DOI:** 10.3389/fnhum.2014.00974

**Published:** 2014-12-03

**Authors:** Roberta Daini, Chiara M. Comparetti, Paola Ricciardelli

**Affiliations:** ^1^Department of Psychology, University of Milano-BicoccaMilan, Italy; ^2^Milan Centre for NeuroscienceMilan, Italy

**Keywords:** face perception, congenital prosopagnosia, unfamiliar face recognition, emotional expressions, non-emotional expression processing

## Abstract

Neuropsychological and neuroimaging studies have shown that facial recognition and emotional expressions are dissociable. However, it is unknown if a single system supports the processing of emotional and non-emotional facial expressions. We aimed to understand if individuals with impairment in face recognition from birth (congenital prosopagnosia, CP) can use non-emotional facial expressions to recognize a face as an already seen one, and thus, process this facial dimension independently from features (which are impaired in CP), and basic emotional expressions. To this end, we carried out a behavioral study in which we compared the performance of 6 CP individuals to that of typical development individuals, using upright and inverted faces. Four avatar faces with a neutral expression were presented in the initial phase. The target faces presented in the recognition phase, in which a recognition task was requested (2AFC paradigm), could be identical (neutral) to those of the initial phase or present biologically plausible changes to features, non-emotional expressions, or emotional expressions. After this task, a second task was performed, in which the participants had to detect whether or not the recognized face exactly matched the study face or showed any difference. The results confirmed the CPs' impairment in the configural processing of the invariant aspects of the face, but also showed a spared configural processing of non-emotional facial expression (task 1). Interestingly and unlike the non-emotional expressions, the configural processing of emotional expressions was compromised in CPs and did not improve their change detection ability (task 2). These new results have theoretical implications for face perception models since they suggest that, at least in CPs, non-emotional expressions are processed configurally, can be dissociated from other facial dimensions, and may serve as a compensatory strategy to achieve face recognition.

## Introduction

Prosopagnosia refers to a category-specific perceptual deficit in face recognition. It can be acquired (i.e., resulting from brain damage, mainly after lesions of occipito-temporal regions; Bodamer, 1947) or congenital (McConachie, 1976). Congenital prosopagnosia (CP) is not caused by brain lesions, but is present from birth, and it occurs along with intact sensory visual abilities and normal intelligence (Behrmann and Avidan, 2005). It has been described as a quite common cognitive disorder, which occurs in 2.47% of the population and almost always runs in families (Kennerknecht et al., 2006). It can be quite dysfunctional given the importance of faces in social life (Behrmann and Avidan, 2005).

Faces, in fact, are among the most important visual stimuli we perceive as they *simultaneously* convey several pieces of important social information. They inform us not only about a person's identity, gender, or age, but also about their mood, emotion, and direction of gaze. Thus, faces can be considered *multi-dimensional* stimuli. Although several behavioral and neuropsychological studies have brought evidence for the existence of cognitive and neural mechanisms dedicated to face perception (Kanwisher et al., 1997, 1999; Posamentier and Abdi, 2003; Kanwisher and Yovel, 2006), still little is known about how these various dimensions are coded and how they are integrated into a single face percept. A first classical distinction has been made between facial expression and facial recognition and identity, which would be processed along two separate routes after an initial stage of visual structural encoding (Bruce and Young, 1986; Kanwisher et al., 1997, 1999; Haxby et al., 2000; Posamentier and Abdi, 2003; Kanwisher and Yovel, 2006). Indeed, it has been reported that prosopagnosic patients with lesions in associative visual cortices, despite their deficit in face recognition can still recognize emotional facial expressions, whereas deficits in expression recognition can occur in patients without prosopagnosia (e.g., Kurucz and Feldmar, 1979; Adolphs et al., 1995), suggesting that expression and identity can be processed independently from each other.

Using fMRI, Haxby et al. (2000) proposed a distributed neural system model for face perception in which face responsive regions were grouped in two systems: the core system that includes areas involved with the visuo-perceptual analysis of a face, and the extended system that includes areas that are involved in the extraction of other information (such as semantics, speech, emotions). Within the core system they emphasize a further distinction between the representation of invariant and changeable aspects of faces. In particular, an important functional and anatomical distinction has been made for the processing of invariant aspects (i.e., eyes, nose, mouth, etc.) and that of changeable aspects of the face (such as eye-gaze direction, facial expression, lip movement, and pre-lexical speech perception), with the former being responsible for the processing of face identity, and the latter being involved in the perception of information that facilitates social interaction and communication (e.g., facial expression).

In the analysis of facial expressions the classical models *implicitly* assume an emotional content (Bruce and Young, 1986; Haxby et al., 2000). However, in everyday life people can show expressions on their faces which do not convey an emotional state. A good example is represented by celebrity impersonators who can mimic the ways in which famous people move their faces. Contrary to facial emotional expressions that are universally recognized and expressed in the same way by all individuals (Ekman and Friesen, 1976), this particular kind of facial expression (called dynamic facial signatures) is idiosyncratic, does not carry an emotional content and provides cues beyond the form of the face (Munhall et al., 2002; O'Toole et al., 2002).

The fact that an observer can quickly and easily recognize in the impersonator's performance the facial mimics of that particular famous actor or politician indicates that we have the ability to extract the identity of a face not only from its invariant aspects (e.g., visual appearance), but also from its changeable aspects (e.g., facial motion and expressions), and even when they do not convey an affective state. People move in unique ways and thus have dynamic facial signatures that perceivers can recognize (Lander et al., 1999). Hence, at least for familiar faces, the person's identity is conveyed both by emotional and non-emotional facial expressions (Hill and Johnston, 2001; Posamentier and Abdi, 2003; Lander and Metcalfe, 2007). Moreover, there is evidence that our brain and cognitive systems can also recognize people both from features and from facial expressions that do not convey an affective state (Knappmeyer et al., 2001). However, there are several outstanding issues regarding the processing of non-emotional facial expressions.

What happens when we perceive expressions that are not emotional (e.g., when somebody pulls his/her face in a meaningless but distinct way)? In keeping with the existing cognitive and neural models, would they be analyzed by the same mechanism and cortical regions underlying the processing of emotional facial expressions? Or instead, would they be processed and perceived as a change in the face invariant features? Although Haxby et al.'s model has been modified to accommodate the recognition of familiar faces thorough the processing of non-emotional facial expression by differentiating the role of visual familiarity from the role of person knowledge (O'Toole et al., 2002; Gobbini and Haxby, 2006), no claim has been made about a possible distinction between emotional and non-emotional facial expression in unfamiliar (unknown) faces.

Recently, it is has been proposed that information about identity could be coded both in the FFA and in the STS. Specifically, the FFA would process static features for both familiar and unfamiliar faces, and the STS, as well as processing emotional facial expression, could also code face identity in the form of dynamic, non-emotional identity signatures (O'Toole et al., 2002). Dynamic information, in fact, contributes to face/person recognition particularly in poor viewing conditions and when invariant facial cues are degraded (Knight and Johnston, 1997; Lander et al., 1999, 2001; Lander and Bruce, 2000). This is because characteristic movements and gestures are reliable cues not only to identity, but also to the recognition of faces of unknown people that have already been seen. In other words, face recognition (i.e., the ability to categorize a face as already seen, although unknown) also relies on changeable features of the face and their dynamic patterns, as does face identity (i.e., the ability to recognize a face as familiar and retrieve our knowledge of it). Lander and Davies (2007) using a face recognition task showed that characteristic motion information could be extracted very rapidly and efficiently when learning a new face, thus suggesting that as a face is learned, dynamic facial information is encoded with its identity and could be used for face recognition also in *unfamiliar* faces.

Although, like acquired prosopagnosic patients (Kurucz and Feldmar, 1979; Tranel et al., 1988; Adolphs et al., 1995; but see also Humphreys et al., 2007), congenital prosopagnosic individuals are indistinguishable from controls in perceiving emotional facial expressions (e.g., Behrmann and Avidan, 2005), very little investigation has been carried out to understand whether in this population non-emotional facial expressions can lead to person recognition, and are dissociable from other facial dimensions (i.e., facial features and emotional facial expressions).

The first evidence suggesting that non-emotional facial expressions could be processed in a specific way, dissociable from emotional facial expressions and other facial features, comes from a study by Comparetti et al. (2011) on typical development individuals (young adults). In this behavioral study both the changeable (emotional and non-emotional expressions) and the invariant (features) aspects of unfamiliar faces were manipulated to investigate a possible new dissociation between emotional and non-emotional facial expressions (i.e., expressions that do not have an affective meaning). Participants were asked to perform a recognition task (2AFC paradigm) and a change detection task, using upright and inverted faces. The faces to be recognized could be either identical to the ones presented in the exposure phase (a face bearing a neutral expression), the same but modified in their internal features, emotional and non-emotional facial expressions, or new faces. Once participants recognized a face as an already seen one, they had to detect whether it was identical to the one previously seen or contained a change. The change could regard the size of the eyes or the mouth (invariant feature manipulation) or the presence or absence of an emotional or a non-emotional facial expressions. The accuracy and RT were measured. It was hypothesized that, if the emotional and non-emotional facial expressions were processed differently, a difference in performance for the three manipulations should emerge. The results showed that each of the three different manipulation conditions had a different impact on the inversion effect (i.e., a decrement in performance that occurs when faces are inverted, thought to reflect a disruption in configural processing and in encoding invariant features; Yin, 1969). In particular, the magnitude of the inversion effect differed in the three manipulations, indicating a difference not only in the processing of the invariant features and the emotional facial expressions, but also a further difference in the processing of non-emotional and emotional facial expressions.

These differences could be due to the fact that although both emotional facial expressions and non-emotional facial expressions convey biological motion, only the former would involve the emotional system (i.e., the extended system in Haxby et al.'s model). Since both types of facial expressions convey dynamic facial information, it is plausible that they are processed by the same area of the core system (i.e., the STS). However, other areas outside the core system could also be involved in processing them, causing the differences between emotional and non-emotional expressions (Gobbini and Haxby, 2006). Thus, it is an open question whether non-emotional facial expressions, which seem to be processed differently both from invariant features and emotional facial expressions, can lead to, or contribute to categorize a face as already seen (i.e., face recognition).

Following our previous study (Comparetti et al., 2011), we made the hypothesis that non-emotional and emotional expressions are processed separately as much as invariant features and changeable aspects.

Important hints come from the study of congenital prosopagnosics, who are impaired at recognizing faces, have difficulties in deriving the configural or holistic relations between face features, but can use facial movement information conveyed by a dynamic face to recognize facial identities (Steede et al., 2007) or to discriminate in a matching task whether two sequentially presented dynamic unfamiliar faces were or not the same identity (Lander et al., 2004). CP individuals, similar to patients affected by acquired prosopagnosia (Busigny and Rossion, 2010), are minimally affected by face inversion and some of them even show a better performance for inverted than for upright faces (the “inversion” superiority effect) (Avidan et al., 2011). Therefore, given that it has been found that in typical development individuals invariant features, emotional and non-emotional facial expressions differ in terms of configural face processing (Comparetti et al., 2011), CP individuals may process non-emotional facial expressions differently than invariant face features, and in the same way as typical development individuals. Moreover, if the processing of non-emotional facial expressions is intact in CP individuals, then it is possible that they use them as cues to facilitate face recognition, thus compensating for their face processing deficits.

The aim of the present study was two-fold. First, we wanted to investigate whether facial expressions that do not convey an affective state (i.e., non-emotional facial expressions) are processed in the same way as emotional facial expressions by congenital prosopagnosic individuals. Second, we wondered whether in CP individuals these expressions could be used as a cue to face recognition given that they should not be, or be less impaired in processing the changeable aspects of a face (Steede et al., 2007). To this end, as in Comparetti et al. (2011), we used the face inversion paradigm and we presented static unfamiliar faces in which one of the following facial aspects was changed: emotional expression; “non-emotional” expression; size of invariant features. Two different tasks were used: a same/different person task (recognition task) and a change detection task. The first task allowed us to test the effect of our manipulations on face recognition processing; whereas the second one was designed to test whether, within the same identity, the change of a specific facial aspect was successfully detected. Moreover, we exploited the face inversion effect as an indicator of underlying perceptual processing. A difference in the magnitude of the face inversion effect for each manipulation in each task would reflect a difference in the processing of face recognition and emotional/non-emotional facial expressions.

## Method

### Participants

The study was conducted in accordance with the ethical standards laid down in the 1964 Declaration of Helsinki and fulfilled the ethical standard procedure recommended by the Italian Association of Psychology (AIP). All experimental protocols were also approved by the ethical committee of the University of Milano-Bicocca. All the participants were volunteers and gave their informed consent to the study.

Six participants (3 F and 3 M; aged between 25 and 45 years old; mean = 35; *SD* = 8.83), who reported in a non-structured interview lifelong difficulties in face recognition and showed impaired performance on tests of face recognition, took part in the study. They were right-handed, had normal or corrected-to-normal vision, and had no neurological or neuropsychological deficit aside from the impairment in face processing.

In order to compare them with a control group, their performance was compared with that of 10 typical development individuals (6 F and 4 M). They did have difficulties in face recognition (self-report) and were matched to the CP group by be age [controls aged between 22 and 49 years old; mean = 33.8; *SD* = 9.55; CPs vs. controls *t*_(14)_ = 0.25; n.s.].

### Assessment of congenital prosopagnosia

Due to the fact that there is an ongoing debate on how to diagnose CP, and on the heterogeneity of the deficit (Schmalzl et al., 2008), in the present study we assessed face perception problems reported by the CP participants by means of more than just one neuropsychological test. The problems reported in a pre-test not structured interview concerned perceived face recognition difficulties, uncertainty in face recognition, prolonged recognition times and the development of compensatory strategies, a pattern compatible with the presence of CP. The presence of CP was further confirmed by comparing the performance of each participant to normative data on three face processing tasks: Benton Facial Recognition Test, TEMA Subtest for memory faces, Cambridge Face Memory Test.

The Benton Facial Recognition Test (BFRT, Benton and Van Allen, 1968; Ferracuti and Ferracuti, 1992), widely used for acquired prosopagnosia, is a test to assess face recognition abilities. For each item, individuals are presented with a target face above six test faces, and they are asked to indicate which of the six images match the target face.

In the TEMA (Reynolds and Bigler, 1995), the subtest for memory faces requires the recognition of target faces from sets of photos of individuals differing in terms of age, gender and ethnic backgrounds, with an increasing number of targets and distracters.

The Cambridge Face Memory Test (CFMT, Duchaine and Nakayama, 2006; Bowles et al., 2009) is the most used and valid test to diagnose CP and it measures face memory (Wilmer et al., 2012); participants learn six unfamiliar target faces, and subsequently are required to recognize them from sets of three faces (one target and two distractor faces). Besides, those faces vary from the learned one (e.g., seen from different viewpoints, with visual noise, etc.). The CFMT test includes two versions based on the orientation of faces, upright and inverted.

Table 1 shows the performance of our experimental group at each test. Inclusion criteria required a pathological performance at least in two out of three tests.

**Table 1:** **CP's demographic information and performances on tests of face recognition**.

**Participants**	**Sex**	**Age**	**BFRT cut off: 40**	**TEMA cut off: 30**	**CFMT cut off: 52**
AG	F	42	36/54^*^	25/41^*^	59/72
AT	F	42	39/54^*^	29/41^*^	51/72^*^
PR	M	45	40/54	25/41^*^	39/72^*^
PT	M	27	36/54^*^	17/41^*^	40/72^*^
CR	F	25	40/54	26/41^*^	36/72^*^
EP	M	27	39/54^*^	32/41	46/72^*^

* *Score falling below the cut off*.

### Stimuli

Stimuli were the same as used in Comparetti et al. (2011). The faces were created from digital photos of real faces by means of Adobe Photoshop and Poser 5.0 software (Curios Lab, Inc., ad e-frontier, Inc., Santa Cruz, CA) as follows. Firstly, by means of Photoshop a completely symmetrical face was created by duplicating just one hemi-face of the original face. Therefore, the left and the right hemi-faces were perfect mirror-images of one another. This ensured that none of the stimuli used contained any intrinsic, unintended asymmetries that could facilitate recognition. Then, the mirror digital photos were imported in a different software program (Poser 5.0) to generate 12 neutral basic stimuli. For every face, external features were almost entirely removed by the software so that face recognition could only be based on the internal features.

**Figure 1:**
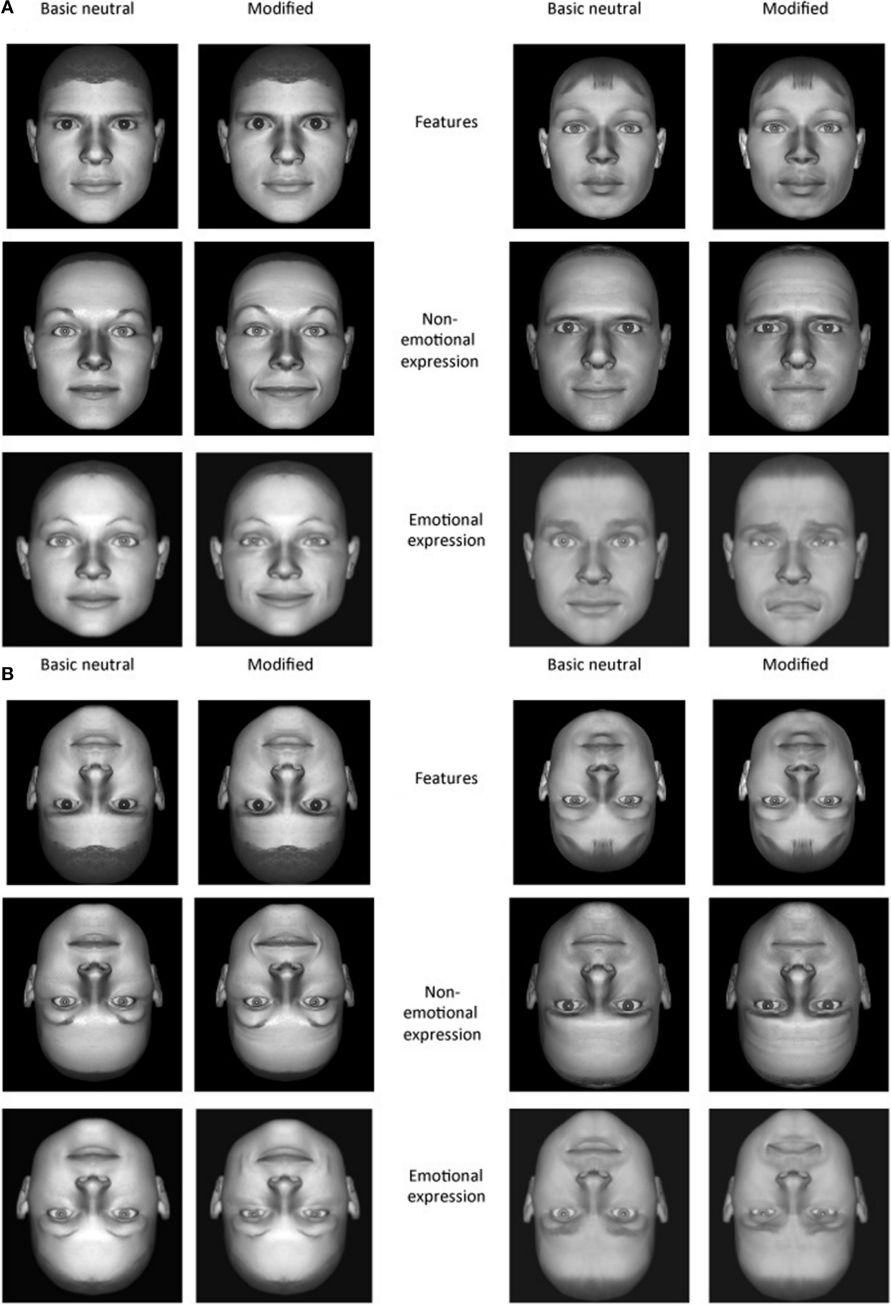
**(A)** Examples of basic neutral faces (male and female) and their modified versions. The changes in features are depicted in the two upper rows. On the left enlarged eye size; on the right enlarged mouth size. The changes in non-emotional expressions are depicted in the middle rows. On the left the change occurred in the upper part of the face; on the right the change occurred in the lower part of the face. The changes in emotional expression version are depicted in the lower rows. On the left a happy expression; on the right a sad expression. **(B)** Stimuli are showed inverted. Each manipulation complied with the parameters of biological compatibility.

The stimuli comprised 12 neutral basic (unmodified) faces generated by Poser and three sets of modified faces in which different manipulations were made (features, emotions, and non-emotional facial expressions). Among the neutral stimuli, 4 were target stimuli (2 picturing females and 2 picturing males) and 8 were distracters (4 F and 4 M), plus 72 modified stimuli which were generated by target and distracter stimuli. For every manipulation, indeed, two different versions of the same manipulation were created (3 different manipulations × 2 versions = 72) using different neutral faces (see Figure 1). The first manipulation, regarded the size of features. From each target stimulus and from each distracter, one modified stimulus (version 1) was created in which the eyes were enlarged and another one was created in which the mouth was enlarged (version 2). Both changes consisted of an increase in size of 1 Poser software unit. This unit respects the boundaries of biological compatibility. The second manipulation, regarded emotional facial expressions. Neutral stimuli were now manipulated by means of Poser 5.0 software to show either a happy (version 1) or a sad (version 2) expression. Finally, the non-emotional facial expressions were created by manipulating the neutral faces in their upper (version 1) and lower part (version 2) respectively, around the eyes and the mouth. In doing so, the resulting facial expressions did not express an affective state (i.e., non-emotional facial expressions).

In order to validate the modified stimuli for use in the present and in other studies (e.g., Comparetti et al., 2011) a scalar rating was performed on a sample of 36 stimuli (12 randomly selected from each stimulus set) to evaluate whether they conveyed or not an emotional facial expression. The selected stimuli were presented in upright orientation on a PC display. Twenty typical development participants who did not take part in the present study (12 F and 8 M, aging between 18 and 32 year old) had to evaluate the faces in a Likert-like scale from 0 (stimulus does not express any emotions) to 4 (stimulus expresses clearly an emotions) (see Table 2). Following that, they had to indicate which emotions they perceived. They could choose among 8 alternatives: happiness, sadness, anger, disgust, fear, surprise, “other,” or “non-emotions.” Each stimulus lasted until response.

**Table 2:** **Mean percentage (%) of emotional and non-emotional ratings given to each type of modified stimulus**.

	**Happiness (%)**	**Sadness (%)**	**Surprise (%)**	**Fear (%)**	**Disgust (%)**	**Anger (%)**	**No emotion/Other (%)**
Emotional expression (happiness)	**90**	0	1	0	0	0	9
Emotional expression (sadness)	0	**72**	0	1	13	6	8
Non-emotional expression (upper part)	4	12	22	3	5	5	**49**
Non-emotional expression (lower part)	2.5	25	4	2.5	2.5	5	**58.5**
Features (enlarged eyes)	2.5	2.5	25	10	4	0	**56**
Features (enlarged mouth)	11	4	16	4	0	6	**59**

*Frequency values of participants answers falling above 50% are in Bold*.

A univariate Analysis of Variance (ANOVA) was performed on the mean percentages of emotional and non-emotional ratings. The effect of stimulus condition was significant [*F*_(1, 19)_ = 81.19; *p* < 0.0001]. The results were as follows. In the case of manipulation of features and manipulation of non-emotional facial expressions the stimuli were generally perceived as not expressing a particular affective state and they did not differ from each other, whereas all the stimuli bearing an emotional facial expression were judged as expressing an emotion and differed both from those with modified features (*p* < 0.0001) and from those displaying a non-emotional facial expression (*p* < 0.0005). Moreover, faces expressing happiness were judged as happy stimuli, and those expressing sadness as sad stimuli. Therefore, the rating analysis corroborated the validity of our face stimuli.

In the present experiment, each face (7.1° × 9.2°) was presented in gray scale and against the same black colored background. All of the stimuli were presented both upright and inverted (see Figure 1).

### Apparatus

The experiment took place in a dark, sound attenuated room. Participants sat in front of a PC computer monitor at a distance of approximately 70 cm. The screen was framed with a circle black carton board of about 15 cm of diameter. Stimulus presentation and registration of task performance were controlled by program Presentation version 9.8. Two keyboards were used: one for the participants, covered by a black card with a hole in correspondence with the button “yes” and “no” (recognition task, see below) and one for the experimenter (same/different task, see below).

### Procedure

The experiment was divided in two sessions, an exposure and an experimental session. In the exposure session the participants saw on the screen the 4 target faces, one by one, 10 times, for 3 s each time. The experimental session followed the exposure one and was divided in four blocks: 2 of upright faces and 2 of inverted faces. In each block neutral and manipulated faces were presented randomly. For each experimental trial the sequence of events was as follows. The trial started with a fixation cross in the center of the screen which lasted 250 ms, then the face stimulus was presented in the center for 500 ms, then there was a gray screen for each task, the same/different person task and the change detection task. For every stimulus participants were asked to indicate whether or not the face was one of the target stimuli. Participants had to press the button “yes” if they saw the face in the exposure phase, or the key no if they did not recognize the face (2 Alternative Forced Choice paradigm). When a stimulus received a “yes” response, participants had then to judge if the stimulus was exactly the same as the one seen in the exposure phase or if there was some change. For the same/different task the experimenter registered the participant's answer on another keyboard pressing the “same” or “different” key. For either the recognition task or the same/different task accuracy was recorded and analyzed.

We used the presence of the inversion effect as a marker of configural processing (e.g., Rossion, 2008).

## Results

The percentage of correct responses was used as a dependent variable (Tables 3, 4).

**Table 3:** **The mean percentages of correct responses for each participant subdivided for each condition in Task 1 (Recognition task)**.

	**Neutral upright**	**Neutral inverted**	**Features upright**	**Features inverted**	**Non-emotional expressions upright**	**Non-emotional expressions inverted**	**Emotional expressions upright**	**Emotional expressions inverted**
**CPGROUP**								
AG	85	85	76.9	80.7	71.4	69.2	84.2	88.5
AT	75	60	76.9	69.2	71.4	50	76.3	65.4
P	65	85	69.2	73.1	85.7	80.7	76.3	88.5
PR	70	65	57.7	57.7	71.4	76.9	47.4	53.8
PT	56.7	60	60	53.9	81	61.5	61.4	46.1
CR	83.3	79.2	68.8	71.9	78.1	71.9	84.4	71.8
**CONTROLS**								
BP	100	87.5	87.5	60.7	86.6	75	81.3	93.8
PV	100	100	100	100	100	100	100	93.8
MD	75	50	68.8	50	50	56.3	50	62.5
SE	100	87.5	100	100	100	100	87.5	100
CF	100	75	93.8	92.9	87.5	86.6	100	93.8
TF	100	100	100	100	100	100	93.8	93.8
FS	100	75	87.5	62.5	80.4	62.5	78.6	62.5
IT	100	87.5	87.5	100	68.8	79.5	65.2	60.7
CA	75	100	87.5	100	87.5	93.8	93.8	87.5
AT	100	71.4	100	67	81.3	68.8	100	68.8

**Table 4:** **The mean percentages of correct responses for each participant subdivided for each condition in Task 2 (Change detection task)**.

	**Neutral upright**	**Neutral inverted**	**Features upright**	**Features inverted**	**Non-emotional expressions upright**	**Non-emotional expressions inverted**	**Emotional expressions upright**	**Emotional expressions inverted**
**CPGROUP**								
AG	88.9	77.8	22.2	17.6	50	0	21.4	20
AT	72.7	64.3	44.4	30	25	28.6	25.9	23.8
EP	45.5	80	12.5	9.1	100	18.2	33.3	38.5
PR	60	55.6	31.6	58.8	75	58.3	55.6	60
PT	52.9	0	35.7	0	50	100	29.2	10
CR	80	100	100	0	88.9	0	9.1	0
**CONTROLS**								
BP	75	50	56.3	26.8	75	62.5	93.8	59.8
PV	87.5	62.5	6.3	0	47.3	25	93.8	31.3
MD	87.5	37.5	43.8	31.3	68.8	56.3	50	68.8
SE	87.5	75	62.5	34.8	92.9	43.8	100	85.7
CF	50	100	31.3	52.7	68.8	39.3	100	72.9
TF	57.1	50	43.8	37.5	87.5	25	93.8	86.6
FS	37.5	25	81.3	93.8	85.7	100	85.7	87.5
IT	62.5	62.5	62.5	25	93.8	79.5	85.7	45.5
CA	87.5	75	43.8	25.9	81.3	43.8	93.8	68.8
AT	75	85.7	25	60.7	75	50	93.8	68.8

An ANOVA was run separately for each task (recognition and change detection) and for each orientation (upright and inverted), with group (CPs and controls) as a between-subject factor and condition (neutral, features, non-emotional, and emotional expressions) as a within-subject factor. *T*-test statistics for independent samples were run as *Post-hoc* tests to compare the performances of the two groups for significant interactions. *T*-test statistics against the null hypothesis (50%) were also performed in order to test that the effects were not due to chance.

All statistical analyses were conducted using the software package Statistica for Windows (version 8.0, Statsoft Inc., 2007). The variances between groups were assessed by Levene's test for the homogeneity of the variances.

**Figure 2:**
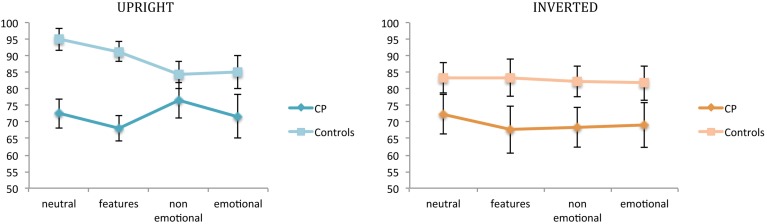
**Mean percentages of correct responses in the recognition task (task 1)**. Percentages are plotted for up-right and inverted presentation as a function of experimental manipulations. Error bars represent standard error of the mean. ^*^Between subjects significant differences.

Figure 2 illustrates participants' performance (CPs and controls) at the first task for each experimental condition.

A first ANOVA was run for the recognition task and the upright condition.

A main effect of group emerged [*F*_(1, 14)_ = 9.72; *p* = 0.007], confirming that the two groups came from different populations in terms of their ability to recognize unfamiliar faces. As expected, controls were better than CPs in recognition (88.87 vs. 72.19%, respectively). However, the significant interaction between group and condition [*F*_(3, 42)_ = 3.196; *p* = 0.033] indicates that this was the case only for neutral faces [controls: 95% vs. CPs: 72.5%, *t*_(14)_ = −4.084; *p* = 0.001; Levene test: *F*_(1, 14)_ = 0.096; *p* = 0.761] and for faces with modified features size [controls: 91.26% vs. CPs: 68.08%, *t*_(14)_ = −4.804; *p* = 0.0002; Levene test: *F*_(1, 14)_ = 0.317; *p* = 0.582]. Both non-emotional facial expressions [controls: 84.21% vs. CPs: 76.51%, *t*_(14)_ = −1.14; n.s.] and emotional facial expressions [controls: 85.02% vs. CPs: 71.66%, *t*_(14)_ = −1.62; n.s.] did not differ between the two groups.

No significant main effect of condition emerged [*F*_(3, 42)_ = 1.21; n.s.].

A second ANOVA was run for the recognition task and the inverted condition.

No significant main effect of group [*F*_(1, 14)_ = 3.361; n.s.], or condition [*F*_(3, 42)_ = 0.306; n.s.] emerged. Their interaction was also not significant [*F*_(3, 42)_ = 0.183; n.s.].

These results are coherent with the idea that in control subjects a configural processing of features is triggered only by upright faces (e.g., Diamond and Carey, 1986), and is compromised in CP individuals (e.g., de Gelder and Rouw, 2000; Behrmann and Avidan, 2005).

In order to assess configural face-specific mechanisms, the face inversion effect was computed as the difference in accuracy between upright and inverted faces, and CP individuals' performance was compared to that of controls for each task by means of an ANOVA. No significant effect occurred [Group: *F*_(1, 14)_ = 0.446; n.s.; Condition: *F*_(3, 42)_ = 0.245; n.s.; Interaction: *F*_(3, 42)_ = 3.391; n.s.].

**Figure 3:**
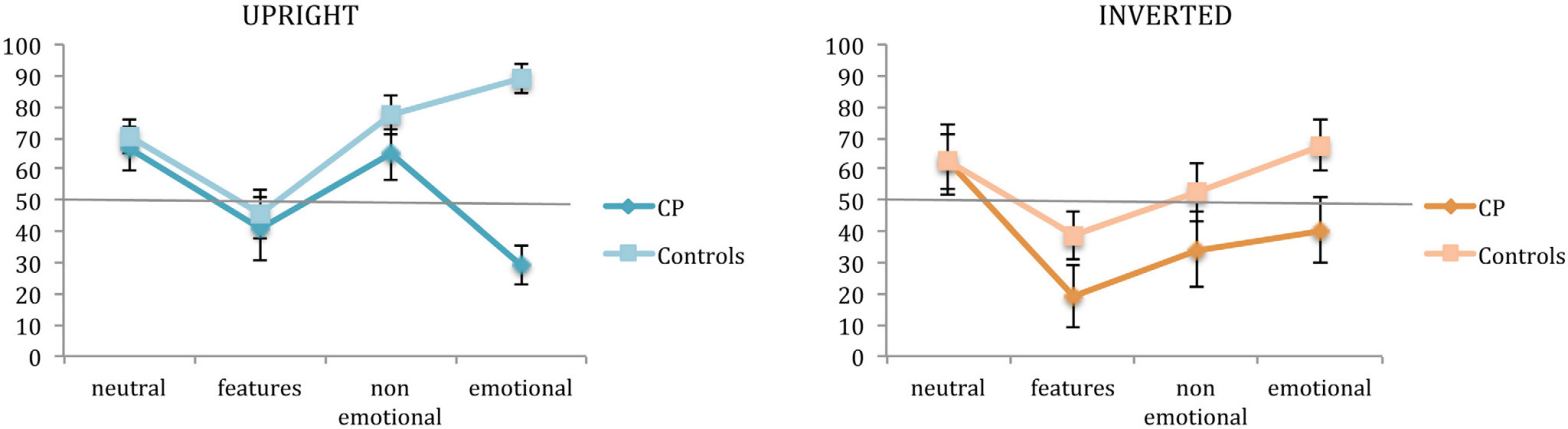
**Mean percentages of correct responses in the change detection task (task 2)**. Percentages are plotted for up-right and inverted presentation as a function of experimental manipulations. Error bars represent standard error of the mean. ^*^Between subjects significant differences.

The detection of features, and non-emotional and emotional expression changes was assessed by the second task, in which participants were requested to judge if the faces recognized as already seen in the first task were exactly the same or somehow different from those seen in the exposure phase. Figure 3 illustrates the performance at the second task of controls and CP individuals for each experimental condition.

A third ANOVA was run on change detection accuracy for the upright condition.

A main effect of group emerged [*F*_(1, 14)_ = 18.68; *p* = 0.0007], confirming a better performance of controls in this task (70.75 vs. 50.41%, respectively). A main effect of condition also emerged [*F*_(3, 42)_ = 5.76; *p* = 0.002], as well as a significant interaction between group and condition [*F*_(3, 42)_ = 6.459; *p* = 0.001]. In particular, these results indicated that the feature condition differed from all the other ones (all *p* < 0.005) and that a difference between the two groups was present only when faces had emotional expressions [controls: 89.04% vs. CPs: 29.08%, *t*_(14)_ = −7.819; *p* < 0.0001; Levene test: *F*_(1, 14)_ = 0.041; *p* = 0.842].

A forth ANOVA was run on change detection accuracy for the inverted condition.

Only the main effect of group [*F*_(1, 14)_ = 5.976; *p* = 0.028] and condition [*F*_(3, 42)_ = 4.199; *p* = 0.01] emerged, confirming a slightly better performance of controls in this task (55.31 vs 39.19%, respectively), and a different performance with feature modified faces than with neutral (*p* = 0.004) and emotional expression faces (*p* = 0.014).

As it can be seen by the inspection of Figure 3, a change in the size of features was really hard to detect both for CP individuals and controls. They all performed below 50%, either with upright or inverted stimuli (CPs: 41.08%, 19.26%, and controls: 45.66%, 38.85%, respectively). This result could be due to the fact that the face processing mechanisms have a low sensitivity to such modifications so as to guarantee efficiency in face identification even when some modifications to the face features (such as a puffiness, for example) occur.

However, the performance at the second task was generally very low in both groups and for this reason we tested each condition in each group vs. the percentage of random responses (50%).

Controls showed a performance above the chance level in the neutral [*t*_(9)_ = 3.618; *p* = 0.006], the non-emotional [*t*_(9)_ = 6.243; *p* = 0.0001] and the emotional expression [*t*_(9)_ = 8.947; *p* < 0.0001] conditions with upright stimuli. As regards the inverted condition, performance was above chance level only in the emotional expression [*t*_(9)_ = 3.051; *p* = 0.014].

In contrast, the CPs' performance was never significantly above the chance level, and in two conditions were significantly lower: features condition of inverted stimuli [*t*_(5)_ = −3.348; *p* = 0.020] and emotional expression condition of upright stimuli [*t*_(5)_ = −3.325; *p* = 0.021].

It is interesting to note that the presence of emotional expressions facilitates the detection of change in the controls, and reduces it in the CPs. It is not the same for non-emotional expressions.

Overall, the results of task 2 suggest a difference in the processing of emotional and non-emotional facial expressions.

**Figure 4:**
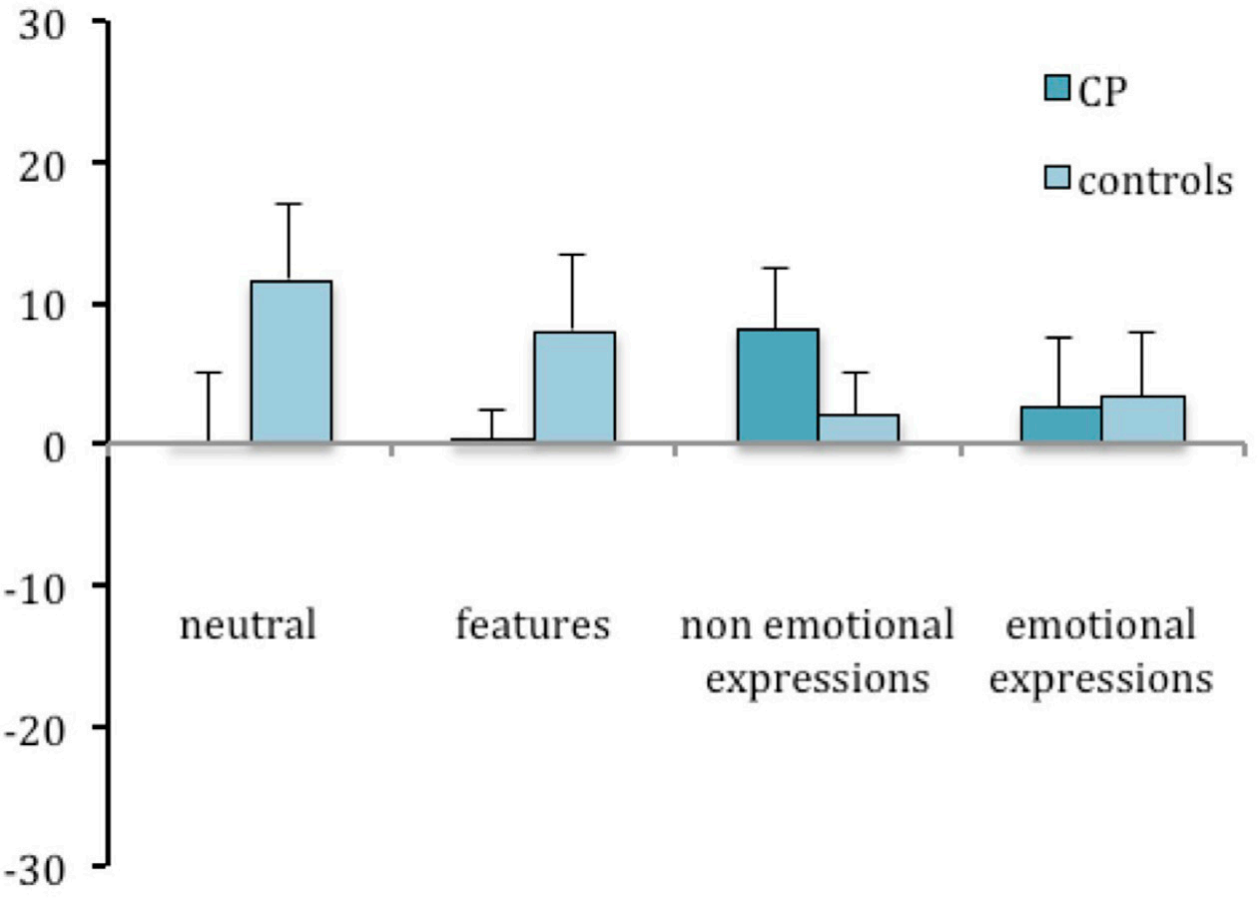
**Mean difference between upright and inverted conditions in the percentages of correct responses in the recognition task (task 1) plotted as a function of group and experimental manipulations**. Error bars represent standard error of the mean.

**Figure 5:**
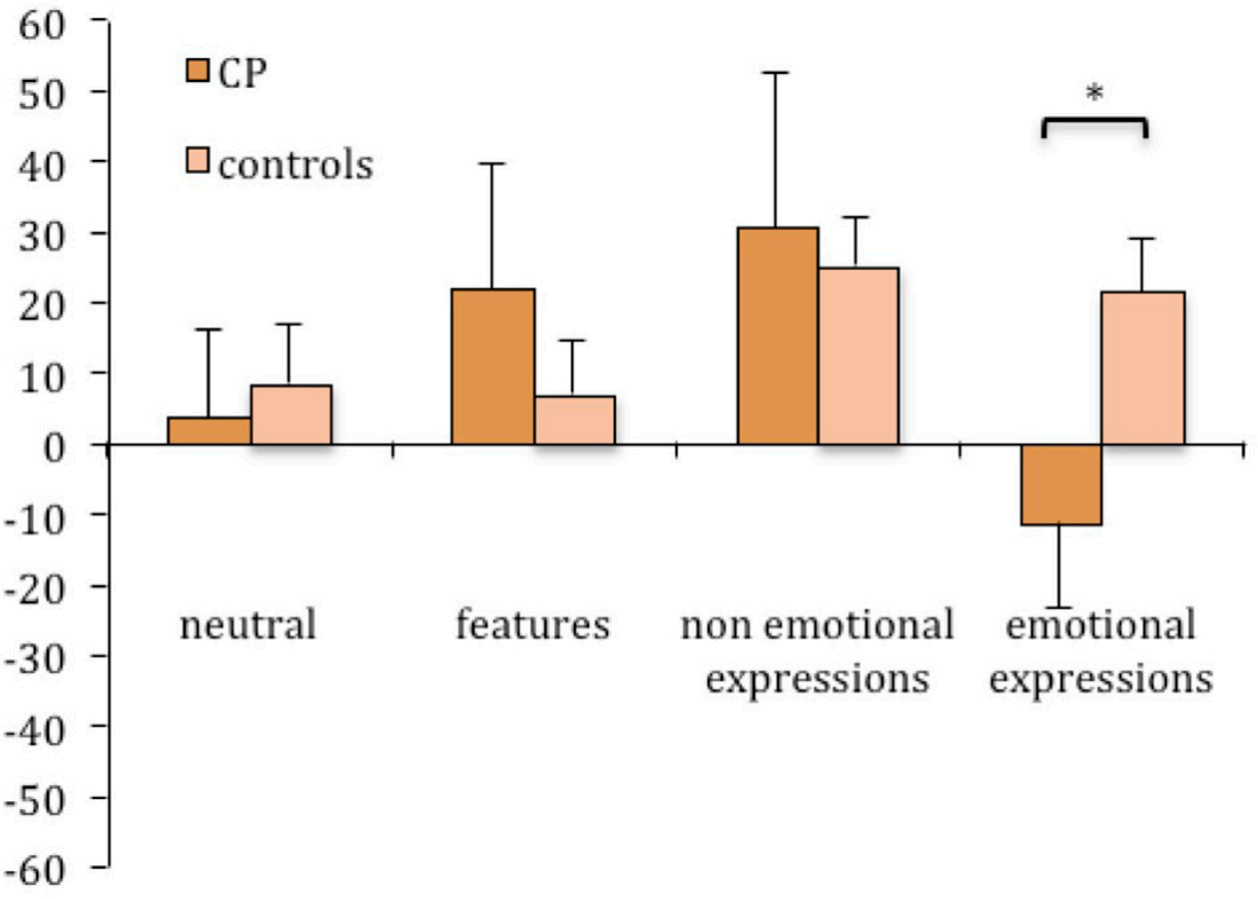
**Mean difference between upright and inverted conditions in the percentages of correct responses in the change detection task (task 2) plotted as a function of group and experimental manipulations**. Error bars represent standard error of the mean. ^*^Between subjects significant differences.

The face inversion effect was computed for task 2 as well, and an ANOVA was run with group as a between-subject factor and condition as a repeated-subject factor. No significant effects were found [Group: *F*_(1, 14)_ = 0.289; n.s.; Condition: *F*_(3, 42)_ = 1.763; n.s.; Interaction: *F*_(3, 42)_ = 1.694; n.s.]. Nevertheless, the inspection of Figures 4, 5 suggests that CP individuals show a greater inversion effect in the condition of non-emotional expressions, in task 2 as much as in task 1.

## Discussion

The aim of this investigation was to shed new light on emotional and non-emotional facial expression processing and to investigate whether in CP individuals these expressions could be used as a cue to face recognition, given that they should be less impaired, or not at all impaired (e.g., Steede et al., 2007). Two consecutive tasks were used. In the first task participants had to recognize static unfamiliar faces which could differ either in the emotional facial expressions, in the non-emotional facial expressions, or in the size of invariant features from a set of previously presented faces. The face stimuli were presented either upright or inverted. We also developed a new task (task 2—change detection task), in which participants were asked to detect whether or not a change occurred in the recognized face compared to the exposure session.

The first main result that emerged from our data was that in task 1, in the upright presentation condition, CPs had a significantly worse performance than controls only for two conditions: neutral and feature-modified faces. This is in line with the hypothesis proposed in the literature (e.g., de Gelder and Rouw, 2000; Behrmann and Avidan, 2005) in which congenital prosopagnosia is characterized by an impairment in processing the invariant features of faces.

The second main result concerned the fact that we did not find a difference between CPs and controls in the recognition of unfamiliar face (as our stimuli were) when the manipulations involved facial expressions (emotional and non-emotional), thus suggesting in CPs both a dissociation between changeable and invariant aspects, and a spared processing of the changeable aspects of the face. Although it has already been shown that dynamic facial expressions can help face recognition (Longmore and Tree, 2013), our finding further indicates that CPs could effectively use non-emotional facial expressions of static images as a cue to recognition, but this seems not to be the case for emotional ones (as evident from their performance in task 2).

Even though we did not formally assess CP abilities in discriminate emotional expressions, it is worth noticing that CP individuals were bad at detecting emotional expression changes but this did not seem to affect their ability to detect a change in non-emotional facial expressions. This result is new and suggests that CPs' good performance in the detection of changes in facial expressions is likely to reflect the use of face motion cues even when they have to be derived from a static image of the face, as in the present study.

The anatomo-functional correlate of the processing of changeable aspects of a face is considered to be the Superior Temporal Sulcus (STS; Haxby et al., 2000), the same area which also underlies the processing of biological motion (Allison et al., 2000). It has been reported that responses to facial expressions and other changeable aspects of the face, such as gaze directions, have different locations in the STS (Engell and Haxby, 2007). Therefore, given the heterogeneity of STS, and on the basis of our results, it could be argued that STS region functionality is preserved in CPs. Therefore, one may expect that CP individuals could also perceive biological motion, in the same way they could process the changeable aspect of a face. Future research is needed to clarify this issue.

Interestingly, our results also bring evidence of a further differentiation between emotional and non-emotional facial expression processing. A new finding that extends the results present in the literature (Longmore and Tree, 2013), and is not accommodated by many face recognition models (Bruce and Young, 1986; Kanwisher et al., 1997, 1999; Haxby et al., 2000; Posamentier and Abdi, 2003; Kanwisher and Yovel, 2006). Although CPs' performance in the recognition task did not differ from that of controls in the emotional and non-emotional expressions conditions, in the second task it dropped severely when the change occurred in the emotional facial expressions.

Taken together these data indicate that the processing of emotional and non-emotional facial expressions differs and that a successful recognition of unfamiliar faces can rely on the detection of non-emotional changeable facial features, at least in subjects affected by CP. A possible explanation for this is that emotions conveyed by facial expression have a more universal meaning than non-emotional facial expressions, which instead can be idiosyncratic and more suitable to face recognition (idiosyncratic dynamic facial signature, as defined by O'Toole et al., 2002). In other words, emotional facial expressions are less useful in recognizing an unfamiliar face which has been seen only once. Hence, non-emotional expressions can be used as a better cue to face identity even when the face is unfamiliar. Note also that our findings demonstrate an accurate detection of non-emotional expressions (task 2), other than a dissociation with emotional expressions. Therefore, they can be memorized and used independently from emotional expressions for correct face recognition both by controls and CPs.

We suggest that CPs could rely more on changeable features for improving face recognition, and this is why they could also be more sensitive to detecting differences in these face dimensions.

Our explanation is consistent with the results from a previous study by Lander and Davies (2007), who claimed the possibility of recognizing faces from facial expression even if they are unfamiliar because as a face is learnt, information about its characteristic motion is encoded with identity. Indeed, it seems that typical development individuals were able to extract and encode dynamic information even when viewing a face for a very short time, such as in our exposure session. Our findings are consistent with this idea and support the proposal of a rapid learning of the characteristic of “implied” motion patterns. In this vein, CPs may have developed a special ability to extract information on the identity from the changeable aspects of faces at the expense of a more fine-tuned emotional expression processing.

In controls, the presence of an emotional expression, in fact, facilitates the detection of a difference in the recognized face, while in CPs the performance associated with these stimuli is greatly reduced (task 2). This indicates that in CPs the affective component of facial expression does not play a key role in face recognition.

In line with O'Toole et al. (2002) model, we propose that the processing of facial changeable aspects can lead to face identification since important cues to identity information are extracted through it. These cues are useful for recognizing both familiar (Albonico et al., 2012) and unfamiliar faces, as shown by previous studies (Longmore and Tree, 2013) and the present study. In particular, we argue that the processing of non-emotional facial expressions is preserved and enhanced in CP individuals, who can then use it to compensate their face recognition deficits. We also speculated that the nature of the processing of the changeable aspects of a face could be configural. Specifically, this is true for non-emotional expressions as it is revealed by the presence of a large inversion effect in CP participants both in the recognition and in the change detection task. Interestingly, in our second task (change detection task) the processing of emotional facial expressions seems to be analytic rather then holistic. In fact, not only did CPs show a very poor performance in the detection of a change in the emotional expressions, but they also showed an “inversion of the inversion effect” (i.e., a better performance for inverted than upright stimuli). This is in line with previous studies (Chen and Chen, 2010), which suggested that relevant information for emotion detection is extracted better by facial single district movements and are processed more analytically than non-emotional expression information.

We think that the configural processing of invariant features is the typical mode to reach face recognition and identification, but when this mechanism is impaired such as in congenital prosopagnosia, the analytic processing of single features and the processing of the non-emotional expressions (which are changeable aspects of a face and are processed via a different and dissociable pathway from that of the facial features) can help compensate for face recognition impairments.

In conclusion, congenital prosopagnosics, even if characterized by a deficit in the global processing of invariant features, could show a preserved analysis of changeable aspects, in particular of non-emotional facial expressions which can be used to face recognition.

A speculative hypothesis, to test in future study with a bigger sample size, could be that, although the configural mechanisms processing invariant features are impaired in CPs (in keeping with their difficulty in face recognition tests), the configural processing of changeable aspects could instead be preserved.

## Author contributions

Roberta Daini designed the experiment and analyzed the data, wrote the manuscript, discussed the results, and prepared the figures. Chiara M. Comparetti designed, performed the experiment, wrote the Method Section and prepared the stimuli. Paola Ricciardelli discussed the results, wrote and revised the manuscript.

### Conflict of interest statement

The authors declare that the research was conducted in the absence of any commercial or financial relationships that could be construed as a potential conflict of interest.
